# Sustained deep molecular responses in patients switched to nilotinib due to persistent *BCR-ABL1* on imatinib: final ENESTcmr randomized trial results

**DOI:** 10.1038/leu.2017.247

**Published:** 2017-09-01

**Authors:** T P Hughes, B Leber, F Cervantes, N Spector, R Pasquini, N C D Clementino, A P Schwarer, P E Dorlhiac-Llacer, F-X Mahon, D Rea, A Guerci-Bresler, S Kamel-Reid, I Bendit, S Acharya, T Glynos, D Dalal, S Branford, J H Lipton

**Affiliations:** 1 Division of Haematology, SA Pathology and South Australian Health and Medical Research Institute, University of Adelaide, Adelaide, South Australia, Australia; 2 Clinical Pathology, McMaster University, Hamilton, Ontario, Canada; 3 Department of Hematology, Hospital Clinic, IDIBAPS, University of Barcelona, Barcelona, Spain; 4 Departamento de Clínica Médica/FM, Universidade Federal do Rio de Janeiro, Rio de Janeiro, Brazil; 5 Division of Hematology and Medical Oncology, Federal University of Paraná, Curitiba, Brazil; 6 Hospital Das Clinicas da UFMG, Belo Horizonte, Brazil; 7 Department of Hematology, Alfred Hospital, Melbourne, Victoria, Australia; 8 Department of Hematology, Hospital das Clinicas FMUSP, São Paulo, Brazil; 9 Laboratoire Hématopoïèse Leucémique et Cible Thérapeutique, Inserm U1035, Université Victor Ségalen, Bordeaux, France; 10 Unité de Thérapie Cellulaire et Clinique Transfusionnelle, Service des Maladies du Sang et EA3518, Hôpital Saint-Louis, Paris, France; 11 Department of Hematology, Brabois Hospital, Vandoeuvre-lès-Nancy, Vandoeuvre, France; 12 Clinical Laboratory Genetics, Genome Diagnostics, University Health Network, Toronto General Hospital, Toronto, Ontario, Canada; 13 Hematology Unit, Faculty of Medicine, University of São Paulo, São Paulo, Brazil; 14 Novartis Healthcare Pvt Ltd, Hyderabad, India; 15 Novartis Pharmaceuticals Corporation, East Hanover, NJ, USA; 16 Leukaemia Unit, Centre for Cancer Biology, SA Pathology, University of South Australia and University of Adelaide, Adelaide, South Australia, Australia; 17 Blood and Marrow Transplant Service, Princess Margaret Cancer Centre, University of Toronto, Toronto, Ontario, Canada

For patients with chronic myeloid leukemia in chronic phase (CML-CP), treatment with a BCR-ABL1 tyrosine kinase inhibitor (TKI), such as imatinib or nilotinib, can result in high response rates and near-normal life expectancies.^1, 2, 3^ Although most patients treated with imatinib achieve a complete cytogenetic response (CCyR) and many achieve a major molecular response (MMR; *BCR-ABL1*⩽0.1% on the International Scale (*BCR-ABL1*^IS^)), MR^4^ (*BCR-ABL1*^IS^⩽0.01%), or MR^4.5^ (*BCR-ABL1*^IS^⩽0.0032%),^4, 5, 6, 7, 8, 9, 10^ results from the Evaluating Nilotinib Efficacy and Safety in Clinical Trials–Newly Diagnosed Patients (ENESTnd) study showed that frontline nilotinib therapy results in higher response rates than frontline imatinib therapy.^9, 10^

ENEST–Complete Molecular Remission (ENESTcmr) was a 48-month, open-label, randomized, phase 3 study designed to investigate whether patients with CCyR but persistent minimal residual disease (MRD) on long-term imatinib could achieve deeper molecular responses by switching to nilotinib 400 mg twice daily vs remaining on imatinib. The study design and methods were previously described in detail^11^ and are summarized in the Supplemental Methods. The study was conducted in accordance with the Declaration of Helsinki. An independent ethics committee or institutional review board at each center approved the protocol. All patients provided written informed consent before randomization. The trial was registered at www.ClinicalTrials.gov (NCT00760877).

Results from the first 24 months of follow-up were previously reported and showed that patients randomized to nilotinib achieved higher rates of MMR and deeper molecular responses than did patients randomized to continue imatinib.^11^ Per the study protocol, patients in the imatinib arm with detectable *BCR-ABL1* at 24 months were allowed to cross over to nilotinib. Here we report updated results from ENESTcmr based on 48 months of treatment for patients who completed the study, including an evaluation of the impact of crossover on patients’ molecular responses.

Of 104 patients randomized to nilotinib 400 mg twice daily, 59 (56.7%) completed 48 months of nilotinib treatment on study and 45 (43.3%) discontinued early (Supplementary Figure 1). Of 103 patients randomized to imatinib, 41 (39.8%) completed 48 months of imatinib treatment on study, 16 (15.5%) discontinued without crossing over to nilotinib, and 46 (44.7%) crossed over to nilotinib (due to detectable *BCR-ABL1* after 24 months of study treatment *(n*=41), imatinib failure *(n*=3), or loss of undetectable *BCR-ABL1* *(n*=2)); after crossing over, 36 patients completed the study on nilotinib and 10 discontinued early. The most common reason for early discontinuation of study treatment in both arms was adverse events (AEs; nilotinib, *n*=19; imatinib, *n*=12 (5 prior to crossover and 7 after crossover); Supplementary Figure 2).

Overall, 56 of 104 patients (53.8%) in the nilotinib arm achieved MR^4.5^ by 48 months; among patients in the imatinib arm, 46 of 103 (44.7%) achieved MR^4.5^ by 48 months, but 13 of these patients achieved MR^4.5^ only after crossover to nilotinib. The Kaplan–Meier–estimated median time to MR^4.5^ was 24 (95% CI, 14.8–47.5) months in the nilotinib arm and was not reached by 48 months in the imatinib arm (Figure 1). Among patients without MR^4.5^ at baseline, 51 of 98 (52.0%) in the nilotinib arm and 40 of 96 (41.7%) in the imatinib arm achieved MR^4.5^ by 48 months (Supplementary Figure 3). Thirty-eight of 46 patients who crossed over from imatinib to nilotinib had not achieved MR^4.5^ on imatinib prior to crossover; of these, 13 achieved a first MR^4.5^ on nilotinib. Of the eight patients who achieved MR^4.5^ prior to crossover (including 4 with MR^4.5^ detected at the time of crossover), seven achieved or maintained MR^4.5^ on nilotinib. Similarly, among the subsets of patients without MMR at baseline or with MMR but without MR^4.5^ at baseline, more patients achieved MR^4.5^ on study with nilotinib than with imatinib (Table 1).

Among patients who crossed over to nilotinib at 24 months and remained on nilotinib with an evaluable molecular assessment at 48 months, *BCR-ABL1*^IS^ levels decreased rapidly following crossover, despite having previously remained relatively stable during imatinib therapy (Supplementary Figure 4). Median (25th–75th percentile) *BCR-ABL1*^IS^ levels decreased from 0.0205% (0.0074–0.1841%) at 24 months to 0.0086% (0.0012–0.0266%) at 48 months in patients who were eligible to cross over at 24 months, crossed over and remained on nilotinib at 48 months. In contrast, *BCR-ABL1*^IS^ levels in patients who remained on imatinib through month 48 despite being eligible to cross over at 24 months were relatively stable; the median (25th–75th percentile) *BCR-ABL1*^IS^ level in these patients was 0.0086% (0.0055–0.0247%) at 24 months and 0.0075% (0.0028–0.0159%) at 48 months.

No patient in either arm progressed to accelerated phase/blast crisis by 48 months. Three patients in each arm died (none due to CML). In the nilotinib arm, two patients discontinued study treatment due to death (one each due to myocardial infarction (on study day 178) and cardiopulmonary failure (day 780)), and one patient died >28 days after study drug discontinuation (due to liver failure (day 1439, ≈8 months after discontinuation)). In the imatinib arm, one patient discontinued study treatment due to death (due to peritoneal carcinomatosis (day 1334)), and two patients died >28 days after study drug discontinuation (one each due to prostate cancer (day 736, ≈18 months after discontinuation) and metastatic non-small cell lung cancer (day 1127, ≈2 months after discontinuation)). No patient who crossed over from imatinib to nilotinib died on study treatment or after study drug discontinuation. The estimated rate of overall survival at 48 months was 96.6% (95% CI, 89.7–98.9%) in the nilotinib arm and 96.9% (95% CI, 90.6–99.0%) in the imatinib arm (regardless of crossover).

Safety results were consistent with the previous analysis.^11^ Additionally, with ≈2 years of nilotinib treatment after crossover, the safety profile of nilotinib 400 mg twice daily in patients who crossed over was comparable to that observed during the first 24 months of study treatment in patients randomized to the nilotinib arm (Supplementary Table 1). Cardiovascular events (CVEs, including ischemic heart disease (IHD), ischemic cerebrovascular events (ICVEs), and peripheral artery disease (PAD)) occurring during study treatment were reported in 13 patients in the nilotinib arm (12.9% (IHD, *n*=4; ICVE, *n*=4; PAD, *n*=7)), two patients in the imatinib arm prior to crossover (1.9% (IHD, *n*=1; ICVE, *n*=1)), and three patients after crossover to nilotinib (6.5% (IHD, *n*=1; ICVE, *n*=1; PAD, *n*=1)). Of the 18 patients in both arms with CVEs during study treatment, 13 had ⩾1 known preexisting cardiovascular risk factor (including age⩾65 years (*n*=9), history of hypertension *(n*=4, all receiving treatment), history of diabetes mellitus (*n*=2, both receiving treatment), and/or history of hypercholesterolemia (*n*=1, untreated)) and/or a prior CVE (*n*=3, including one patient with a history of transient ischemic attack; one patient with a history of thrombosis, deep vein thrombosis, peripheral vascular disorder and transient ischemic attack; and one patient with a history of Raynaud phenomenon) at enrollment. AEs of pancreatitis were reported in 3 (3.0%) and 0 patients in the nilotinib and imatinib arms, respectively; pancreatitis was not reported after crossover in any patient.

Although this study was not powered to evaluate differences in long-term outcomes between the study arms, the results reported here demonstrate that switching to nilotinib therapy may enable some patients with persistent MRD on long-term imatinib to achieve further reductions in *BCR-ABL1* levels, resulting in increased rates of deep molecular response. Moreover, as achievement of a sustained deep molecular response is a key eligibility criterion for attempting treatment-free remission (TFR),^12^ these results suggest that switch to nilotinib may enable some patients to become eligible for TFR and support further investigation of TFR following nilotinib therapy. Importantly, however, not all patients were able to achieve deep molecular responses after switching to nilotinib treatment, and some discontinued nilotinib due to AEs. Thus, both the potential for improved efficacy with nilotinib and the potential for new AEs, including CVEs, should be considered when evaluating whether to switch treatment for a patient. Nonetheless, the observed dynamics of changes in *BCR-ABL1* transcript levels after patients in the imatinib arm crossed over to nilotinib provide a further illustration of the benefits of switching to nilotinib. *BCR-ABL1* levels rapidly decreased following crossover to nilotinib, enabling many patients to achieve MR^4.5^, whereas patients who were eligible to cross over for persistent detectable MRD at 24 months (by which time such patients had received imatinib for a total duration of ⩾4–5 years, including treatment prior to study enrollment^11^) but remained on imatinib had relatively stable transcript levels between 24 and 48 months. These results suggest that most patients have stable *BCR-ABL1* levels following long-term imatinib therapy and are unlikely to achieve further substantial reductions in transcript levels with continued imatinib therapy. Overall, results from ENESTcmr support switching to nilotinib for some patients with persistent MRD after long-term imatinib.

## Figures and Tables

**Figure 1:**
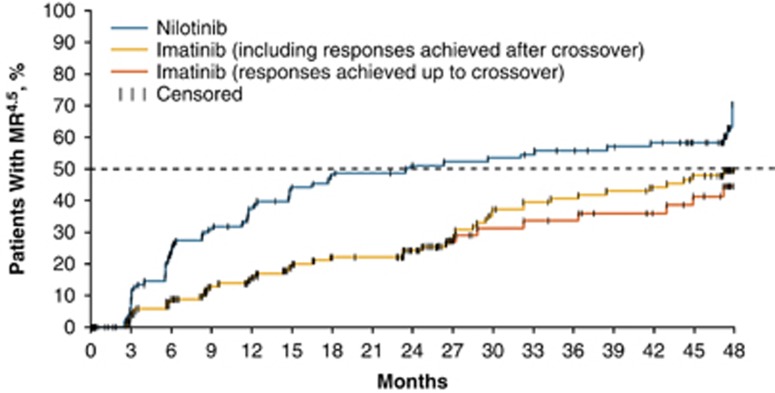
Time to Achievement of First MR^4.5^ (ITT population). ITT, intention-to-treat; MR^4.5^, *BCR-ABL1* ⩽0.0032% on the International Scale.

**Table 1:** Achievement of MR^4.5^ according to response status at baseline and crossover

	*Nilotinib arm*	*Imatinib arm*	P-*value*^a^
*All patients*	*n*=104	*n*=103	
Achieved MR^4.5^ by 48 months			
ITT, *n* (%)	56 (53.8)	46 (44.7)	0.1766
Up to crossover, *n* (%)	56 (53.8)	33 (32.0)	0.0011
After crossover, n/m (%)^b^	NA	20/46 (43.5)^c^	NA
			
*Patients without MMR at baseline*	*n*=24	*n*=28	
Achieved MR^4.5^ by 48 months			
ITT, *n* (%)	8 (33.3)	6 (21.4)	0.4279
Up to crossover, *n* (%)	8 (33.3)	1 (3.6)	0.0055
After crossover, n/m (%)^d^	NA	5/17 (29.4)^e^	NA
			
*Patients with MMR but without MR*^4.5^ *at baseline*	*n*=74	*n*=68	
Achieved MR^4.5^ by 48 months			
ITT, *n* (%)	43 (58.1)	34 (50.0)	0.2273
Up to crossover, *n* (%)	43 (58.1)	26 (38.2)	0.0084
After crossover, n/m (%)^f^	NA	14/28 (50.0)^g^	NA

Abbreviations: ITT, intention-to-treat; MMR, major molecular response; MR^4.5^, *BCR-ABL1* ⩽0.0032% on the International Scale.

a *P*-values are nominal. No multiplicity adjustments were made; therefore, statistical interpretation should be made with caution. *P*-values were obtained using the Cochran–Mantel–Haenszel test.

b Denominator is the number of patients who crossed over.

c A total of 20 patients achieved MR^4.5^ after crossover, including 13 who achieved MR^4.5^ for the first time on nilotinib, 4 who achieved MR^4.5^ on imatinib and maintained the response on nilotinib and 3 who regained MR^4.5^ on nilotinib after losing the response prior to crossover.

d Denominator is the number of patients without MMR at baseline who crossed over.

e Of the 11 patients without MMR at baseline who did not crossover, 1 achieved MR^4.5^ on imatinib and was not eligible to crossover; the other 10 patients were eligible to crossover but did not, and none of these achieved MR^4.5^.

f Denominator is the number of patients with MMR but without MR^4.5^ at baseline who crossed over.

g Of the 68 patients with MMR but without MR^4.5^ at baseline, 28 crossed over and 40 did not crossover. Twenty of the 40 patients who did not crossover achieved MR^4.5^ on imatinib by the end of the study. Fourteen of the 28 patients who crossed over achieved MR^4.5^ by the end of the study, including 10 patients who achieved MR^4.5^ for the first time after crossover and 4 patients who achieved MR^4.5^ before and after crossover.
