# Supplementary material for: Sustained deep molecular responses in patients switched to nilotinib due to persistent BCR-ABL1 on imatinib: final ENESTcmr randomized trial results
Source: Leukemia. 2017 Sep 1;31(11):2529–31. doi: 10.1038/leu.2017.247 (PMC5668492; doi:10.1038/leu.2017.247)
Supplement: Supplementary Information [file leu2017247x1.docx]

**Supplemental methods**

**Study design and treatments**

Evaluating Nilotinib Efficacy and Safety in Clinical Trials–Complete Molecular Remission (ENESTcmr) was a 48-month, open-label, randomized, phase 3 treatment study conducted at 30 sites in Argentina, Australia, Brazil, Canada, France, and Spain (enrollment period: June 2009 through June 2010); the study design and methods were previously described in detail.^1^ Briefly, patients with Philadelphia chromosome–positive chronic myeloid leukemia in chronic phase (CML-CP) and complete cytogenetic response but persistent minimal residual disease (defined as detectable *BCR-ABL1* in 2 consecutive real-time quantitative polymerase chain reaction [RQ-PCR] assessments ≥ 8 weeks apart, in local laboratories, including ≥ 1 assessment within 3 months of randomization) after ≥ 2 years of imatinib 400 to 600 mg daily were randomized by an Interactive Web Response System 1:1 to switch to nilotinib 400 mg twice daily or to continue the same dose of imatinib. Randomization was stratified by the duration of prior imatinib therapy (≤ 36 months or > 36 months) and prior interferon therapy (none, ≤ 12 months, or > 12 months). Patients with a major toxicity ≤ 3 months prior to study entry or a dose modification ≤ 6 months prior to study entry were not eligible for enrollment.

To achieve 80% power to detect a difference of 18% between the 2 arms in the rate of confirmed undetectable *BCR-ABL1* by 12 months (primary endpoint), with a significance level of 0.05 using a two-sided Cochran-Mantel-Haenszel test, enrollment of 85 patients per arm was needed, assuming a response rate of 32% with nilotinib and 14% with imatinib. To account for possible variations in response rates among study strata (allowing the rate in the imatinib arm to range from 10% to 14% across the strata, with the average difference between arms to be as low as 16%, and maintaining a constant odds ratio of 2.89 between the arms), a total enrollment of 96 patients per arm was needed.

Patients in the imatinib arm with detectable *BCR-ABL1* at 24 months were permitted to cross over to nilotinib 400 mg twice daily. Patients in the imatinib arm were also permitted to cross over to nilotinib 400 mg twice daily at any time due to treatment failure or confirmed loss (in 2 consecutive assessments) of either major molecular response (MMR; *BCR-ABL1* ≤ 0.1% on the International Scale [*BCR-ABL1*^IS^]) or undetectable *BCR-ABL1*. Crossover from nilotinib to imatinib was not permitted for any reason. The primary endpoint was not met, as previously described.^1^ Secondary and exploratory endpoints included rates of MR^4.5^ (*BCR-ABL1*^IS^ ≤ 0.0032%), overall survival, time to response, and safety. As of the data cutoff date for the current analysis (July 8, 2014), all patients had completed 48 months of treatment or discontinued early.

**Monitoring**

Molecular responses were assessed by RQ-PCR at an IS-standardized central reference laboratory in Adelaide, Australia, and expressed as the ratio of *BCR-ABL1* to *BCR*.^2^ Molecular responses were assessed at screening/baseline, every 3 months during study treatment, at crossover, and at discontinuation from the study. Hematologic and biochemical laboratory abnormalities were evaluated locally at screening/baseline, every 3 months during study treatment, at crossover, and at discontinuation. Cardiovascular events were defined as ischemic heart disease, ischemic cerebrovascular events, and peripheral artery disease. Modifiable cardiovascular risk factors such as hypertension and diabetes were not assessed at baseline per protocol; however, patient-reported medical history was used to assess the prevalence of these risk factors at baseline.

**Analysis populations and statistics**

The intent-to-treat (ITT) population included all randomized patients. Rates of MR^4.5^ were evaluated in the ITT population and in subsets defined based on molecular response levels at baseline. In the imatinib arm, outcomes and events occurring after crossover were analyzed in the subset of patients who crossed over to nilotinib at any time or crossed over at 24 months due to detectable *BCR-ABL1*; *BCR-ABL1* levels in the imatinib arm were also analyzed in the subset of patients who remained on imatinib through month 48 despite persistent detectable *BCR-ABL1* at 24 months. For analyses of events up to crossover in the imatinib arm, all patients (n = 103) were included; however, for patients who crossed over, only events that happened at or before crossover were included in these analyses.

The safety population included all patients who received ≥ 1 dose of study drug. In the imatinib arm, safety results were evaluated separately for events occurring during imatinib therapy (in all patients) and events occurring during nilotinib therapy (in patients who crossed over).

Rates of overall survival and the median time to MR^4.5^ were estimated using the Kaplan-Meier method. *P* values are nominal, post hoc, and provided for descriptive purposes only; no multiplicity adjustments were made, and statistical interpretation should be made with caution.

**Supplemental data**

**Supplemental Figure 1. Patient status at end of study.**

**
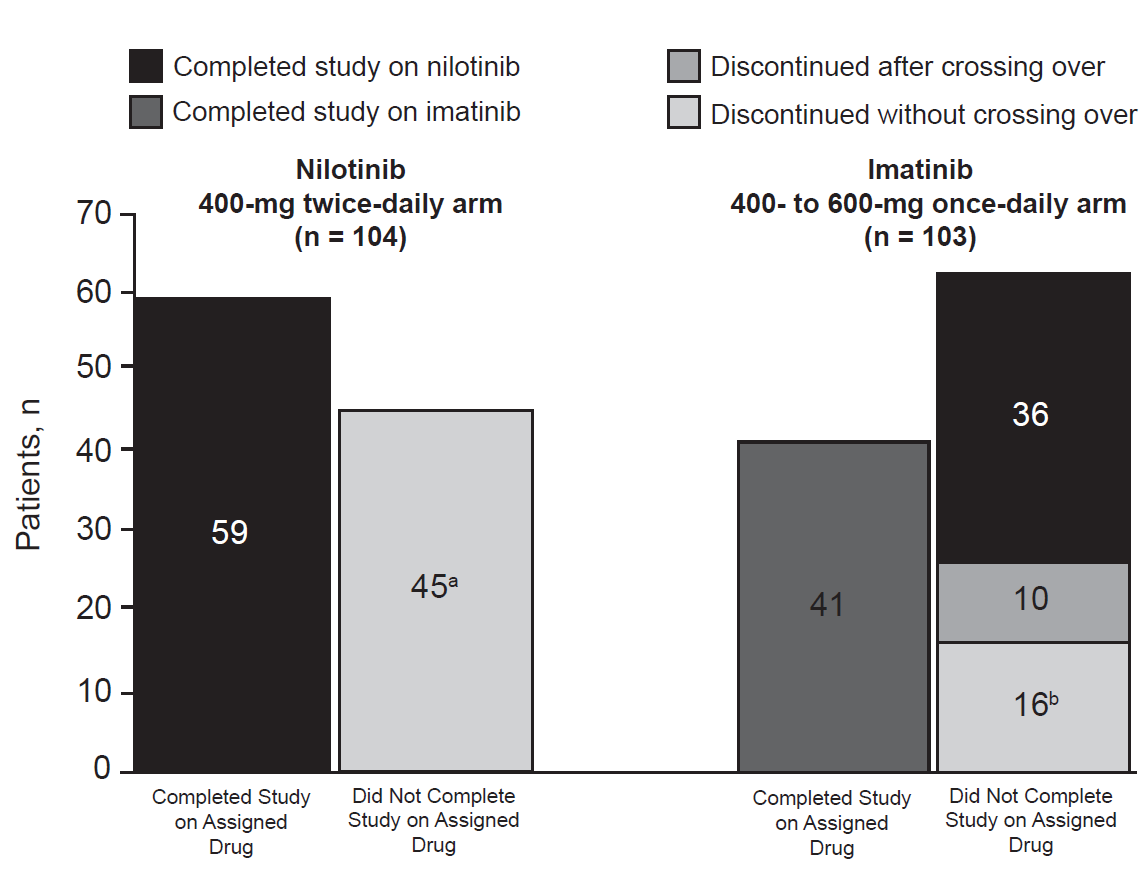
**

^a^ Includes 3 patients in the nilotinib arm who died on study (death was the reason for discontinuation of study treatment for 2 patients; 1 patient died > 28 days after discontinuing study treatment).

^b^ Includes 3 patients in the imatinib arm who died on study (death was the reason for discontinuation of study treatment for 1 patient; 2 patients died > 28 days after study drug discontinuation).

**Supplemental Figure 2. CONSORT diagram.**

**
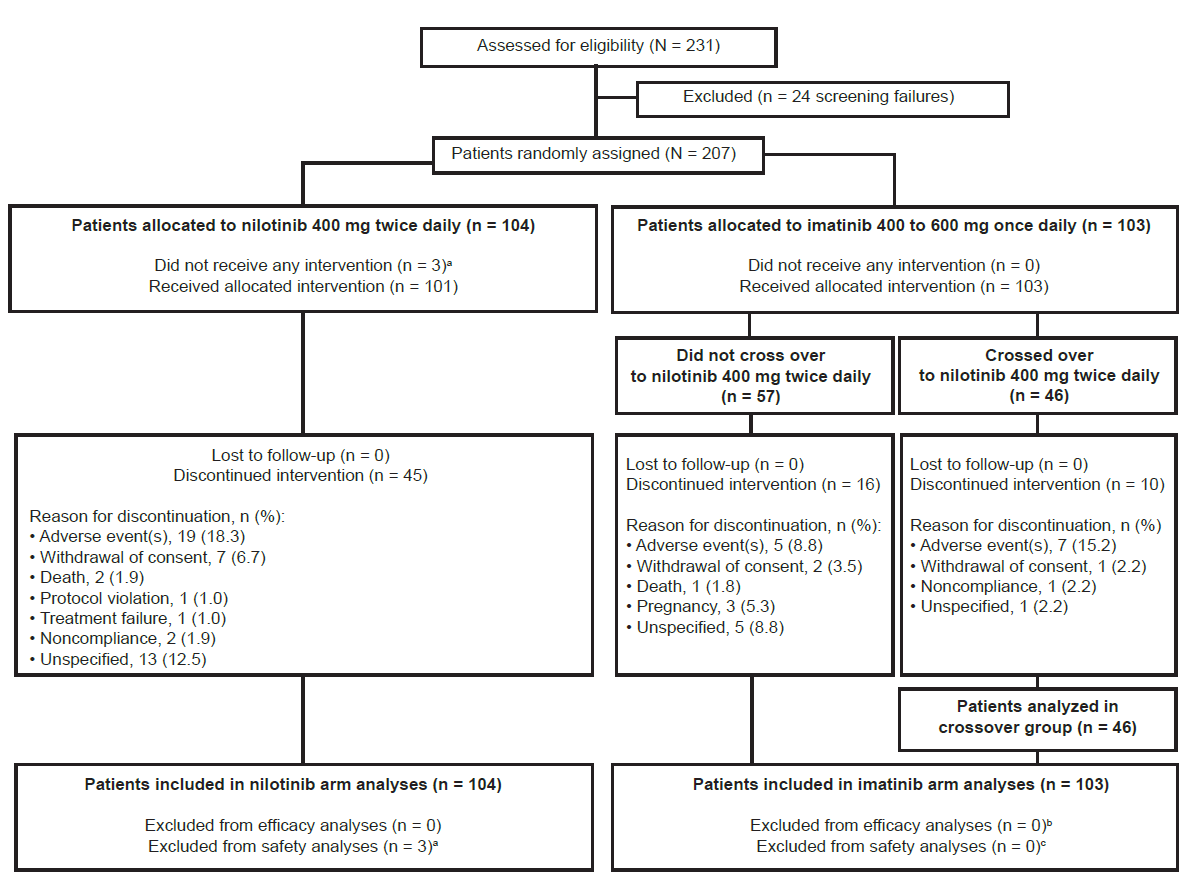
**

^a^ These 3 patients were excluded from the safety analysis because they never received study drug.

^b^ Analyses of events occurring up to crossover and of outcomes in the intent-to-treat population regardless of crossover were evaluated among all randomized patients (n = 103).

^c^ For patients who crossed over, analyses of imatinib safety considered only adverse events occurring prior to crossover, and analyses of nilotinib safety considered only adverse events occurring after crossover.**Supplemental Figure 3. Cumulative incidence of MR^4.5^ over time among patients without MR^4.5^ at baseline.**

**
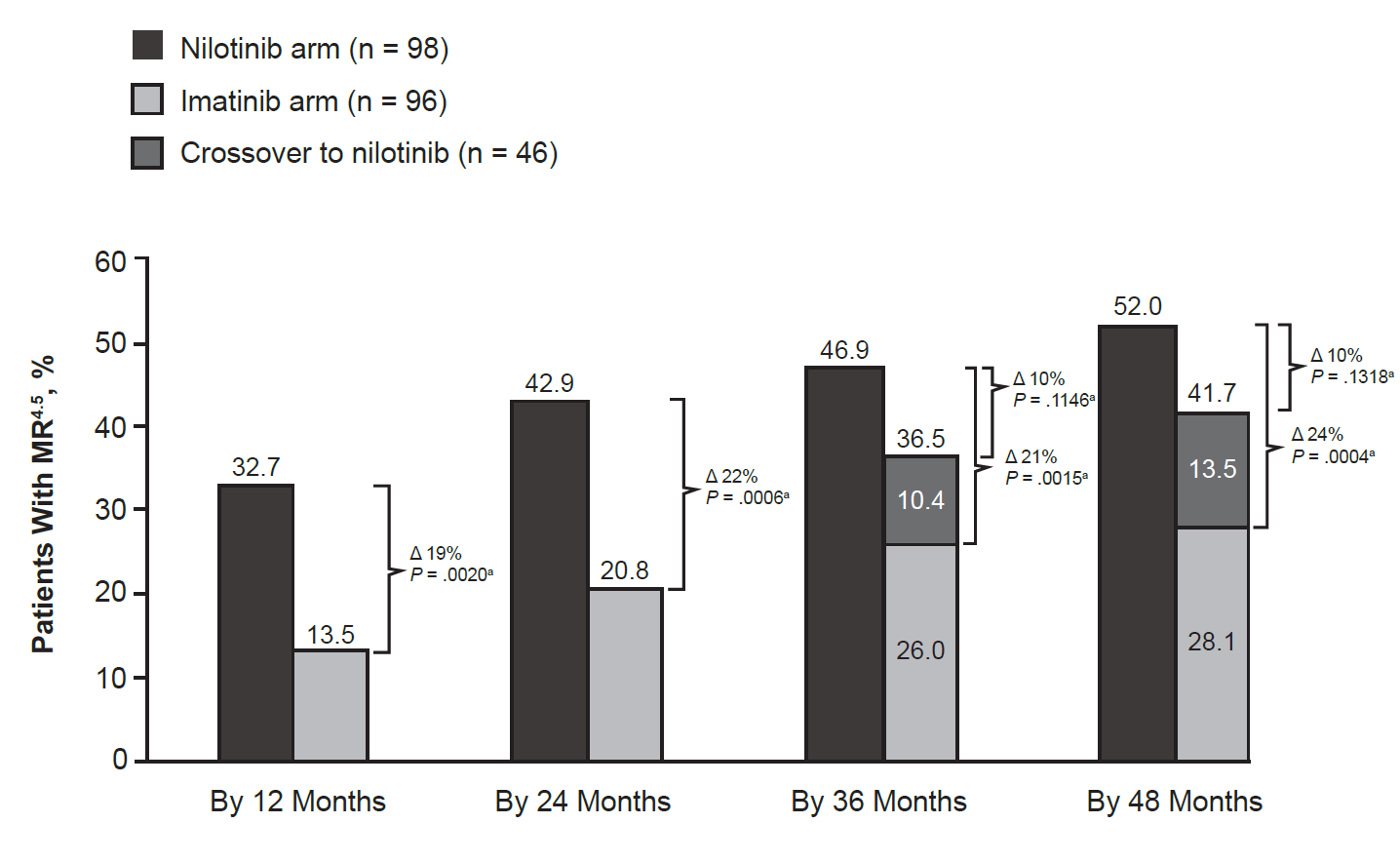
**

MR^4.5^, *BCR-ABL1* ≤ 0.0032% on the International Scale.

^a^ *P* values are nominal and are provided for descriptive purposes only. *P* values were obtained using the Cochran-Mantel-Haenszel test.

**Supplemental Figure 4. Molecular responses over time.** A) *BCR-ABL1*^IS^ levels^a^ over time in patients in the imatinib arm who (A) crossed over to nilotinib at 24 months and remained on study at 48 months (n = 35) or (B) had detectable *BCR-ABL1* at 24 months and remained on imatinib through 48 months (n = 23).

**A)**

**
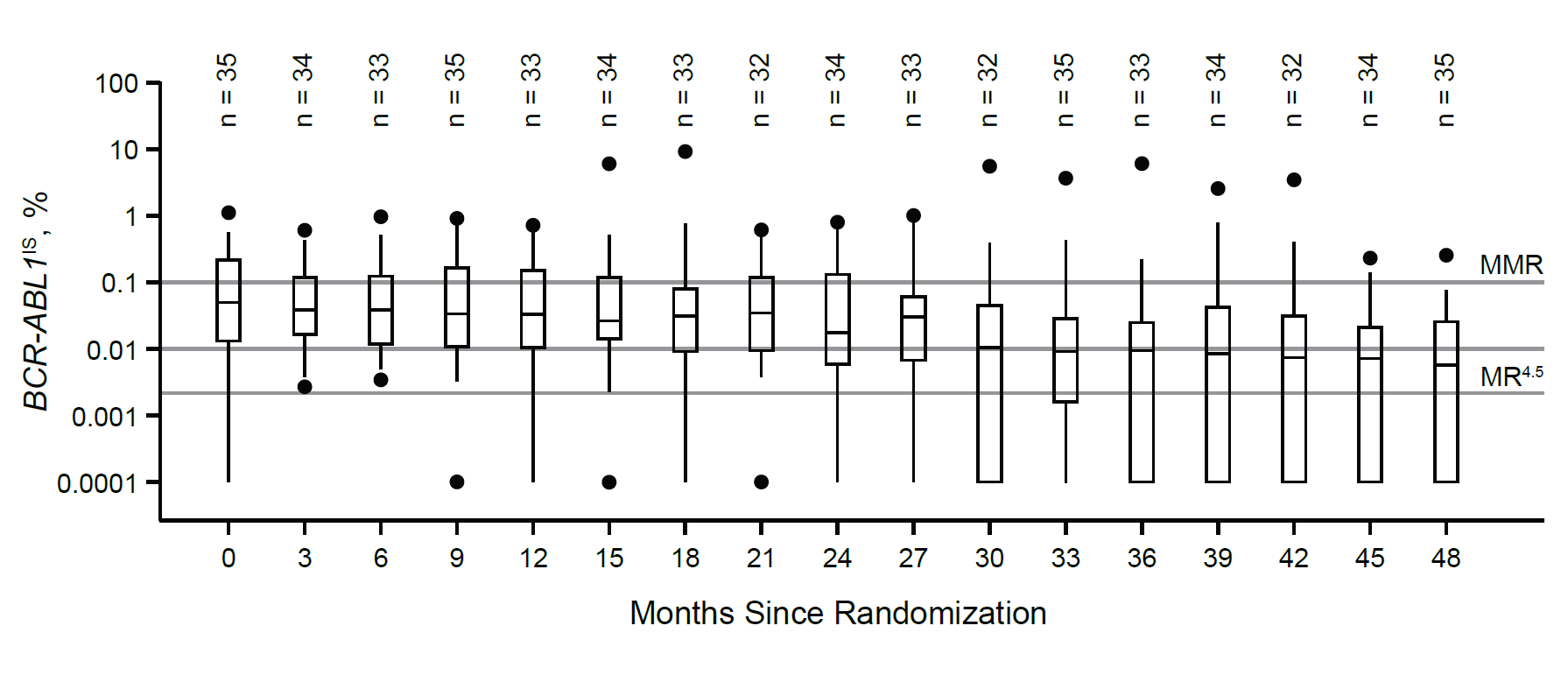
**

**B)**

**
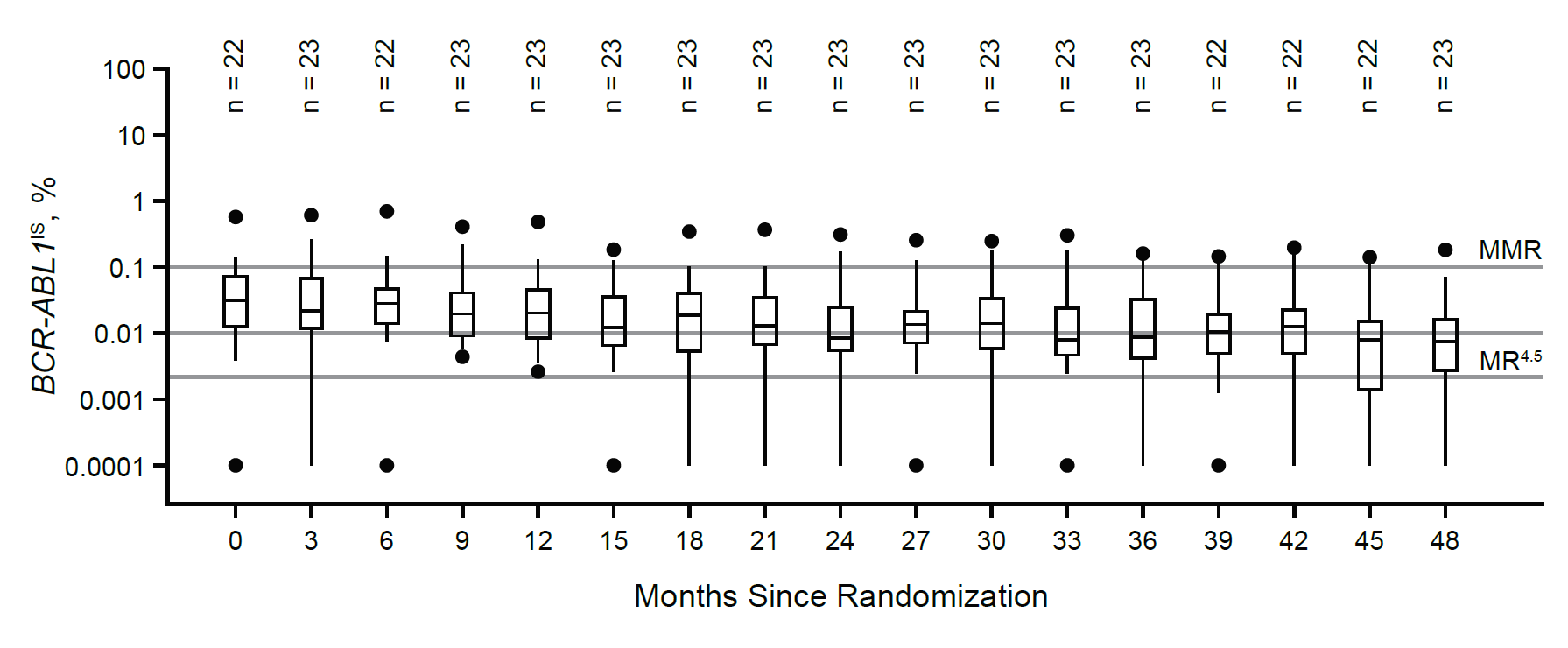
**

IS, International Scale; MMR, major molecular response (*BCR-ABL1*^IS^ ≤ 0.1%); MR^4.5^, *BCR-ABL1*^IS^ ≤ 0.0032%.

^a^ Patient samples with undetectable transcripts are plotted with a *BCR-ABL1*^IS^ value of 0.0001%. Data are shown as box-and-whisker plots, with the boxes representing the 25th percentile, median, and 75th percentile; the whiskers representing 1.5-fold the width of the interquartile range; and the dots representing outliers.

**Supplemental Table 1. Most common drug-related nonhematologic adverse events and newly occurring or worsening laboratory abnormalities**

|  | **Nilotinib**  **400 mg twice daily**  **(n = 101)**  *Median time on nilotinib on study:  47.2 months* | | **Imatinib**  **400 to 600 mg  once daily**  **(n = 103)^a^**  *Median time on imatinib on study: 28.3 months* | | **Crossover to nilotinib**  **400 mg twice daily**  **(n = 46)^b^**  *Median time on study treatment: 25.3 months imatinib; 21.8 months nilotinib* | |
| --- | --- | --- | --- | --- | --- | --- |
|  | **All grades** | **Grade 3/4** | **All  grades** | **Grade  3/4** | **All  grades** | **Grade  3/4** |
| **Drug-related nonhematologic adverse events reported in ≥ 10% of patients in either arm, n (%)** | | | | | | |
| Headache | 34 (33.7) | 2 (2.0) | 3 (2.9) | 0 | 6 (13.0) | 2 (4.3) |
| Rash | 30 (29.7) | 1 (1.0) | 2 (1.9) | 0 | 6 (13.0) | 0 |
| Pruritus | 28 (27.7) | 1 (1.0) | 0 | 0 | 2 (4.3) | 0 |
| Abdominal pain^c^ | 16 (15.8) | 1 (1.0) | 6 (5.8) | 0 | 3 (6.5) | 0 |
| Dry skin | 16 (15.8) | 0 | 0 | 0 | 3 (6.5) | 0 |
| Fatigue | 15 (14.9) | 1 (1.0) | 4 (3.9) | 0 | 5 (10.9) | 1 (2.2) |
| Nausea | 15 (14.9) | 0 | 14 (13.6) | 0 | 3 (6.5) | 0 |
| Muscle spasms | 15 (14.9) | 0 | 17 (16.5) | 0 | 0 | 0 |
| Myalgia | 13 (12.9) | 1 (1.0) | 1 (1.0) | 0 | 5 (10.9) | 1 (2.2) |
| Diarrhea | 6 (5.9) | 0 | 14 (13.6) | 1 (1.0) | 2 (4.3) | 0 |
| **Newly occurring or worsening grade 3/4 hematologic abnormalities, n (%)** | | | | | | |
| Neutropenia | 2 (2.0) | | 7 (6.8) | | 0 | |
| Anemia | 2 (2.0) | | 1 (1.0) | | 1 (2.2) | |
| Lymphopenia | 1 (1.0) | | 1 (1.0) | | 1 (2.2) | |
| Leukopenia | 0 | | 3 (2.9) | | 0 | |
| Thrombocytopenia | 0 | | 3 (2.9) | | 0 | |
| **Newly occurring or worsening grade 3/4 biochemical abnormalities, n (%)^d^** | | | | | | |
| Lipase | 17 (16.8) | | 5 (4.9) | | 3 (6.5) | |
| Phosphate | 12 (11.9) | | 18 (17.5) | | 2 (4.3) | |
| Alanine aminotransferase | 3 (3.0) | | 1 (1.0) | | 0 | |
| Total bilirubin | 3 (3.0) | | 0 | | 1 (2.2) | |
| Magnesium | 2 (2.0) | | 2 (1.9) | | 0 | |
| Creatinine | 1 (1.0) | | 0 | | 0 | |
| Potassium | 0 | | 1 (1.0) | | 0 | |

^a^ Adverse events occurring up to the time of crossover. In the imatinib arm, the median time on imatinib treatment on study among all patients (n = 103) was 28.3 months (47.4 and 25.3 months in patients who remained on imatinib [n = 57] and patients who crossed over to nilotinib [n = 46], respectively).

^b^ Adverse events occurring after crossover to nilotinib.

^c^ Includes patients with adverse events reported as “abdominal pain” and/or “abdominal pain, upper.”

^d^ The study protocol called for routine monitoring of alanine aminotransferase, alkaline phosphatase, amylase, aspartate aminotransferase, calcium, creatinine, lipase, magnesium, phosphorus, potassium, total bilirubin, total protein, and urea levels.

**References**

1. Hughes TP, Lipton JH, Spector N, Cervantes F, Pasquini R, Clementino NCD*, et al*. Deep molecular responses achieved in patients with CML-CP who are switched to nilotinib after long-term imatinib. *Blood* 2014; **124**: 729-36.

2. Branford S, Hughes TP, Rudzki Z. Monitoring chronic myeloid leukaemia therapy by real-time quantitative PCR in blood is a reliable alternative to bone marrow cytogenetics. *Br J Haematol* 1999; **107**: 587-99.
